# Doris Duke Charitable Foundation Fund to Retain Clinical Scientists: innovating support for early-career family caregivers

**DOI:** 10.1172/JCI166075

**Published:** 2022-12-01

**Authors:** Reshma Jagsi, T. DeLene Beeland, Kevin Sia, Lauren A. Szczygiel, Matthew R. Allen, Vineet M. Arora, Megan Bair-Merritt, Melissa D. Bauman, Hillary R. Bogner, Gail Daumit, Esa Davis, Angela Fagerlin, Daniel E. Ford, Rasheed Gbadegesin, Kathy Griendling, Katherine Hartmann, S. Susan Hedayati, Rebecca D. Jackson, Susan Matulevicius, Michael J. Mugavero, Eric J. Nehl, Tuhina Neogi, Judith G. Regensteiner, Michael A. Rubin, Doris Rubio, Kanakadurga Singer, Brownsyne Tucker Edmonds, Anna Volerman, Sandra Laney, Carrie Patton, Sindy Escobar Alvarez

**Affiliations:** 1 Emory University, Atlanta, Georgia, USA.; 2 InsightMed Communications, Asheville, North Carolina, USA.; 3 Doris Duke Charitable Foundation, New York, New York, USA.; 4 University of Michigan, Ann Arbor, Michigan, USA.; 5 Indiana University School of Medicine, Indianapolis, Indiana, USA.; 6 University of Chicago Pritzker School of Medicine, Chicago, Illinois, USA.; 7 Boston University School of Medicine, Boston, Massachusetts, USA.; 8 University of California Davis, Davis, California, USA.; 9 University of Pennsylvania Perelman School of Medicine, Philadelphia, Pennsylvania, USA.; 10 Johns Hopkins School of Medicine, Baltimore, Maryland, USA.; 11 University of Pittsburgh School of Medicine, Pittsburgh, Pennsylvania, USA.; 12 University of Utah Spencer Fox Eccles School of Medicine, Salt Lake City, Utah, USA.; 13 Duke University School of Medicine, Durham, North Carolina, USA.; 14 Vanderbilt University, Nashville, Tennessee, USA.; 15 University of Texas Southwestern Medical Center, Dallas, Texas, USA.; 16 The Ohio State College of Medicine, Columbus, Ohio, USA.; 17 University of Alabama Heersink School of Medicine, Birmingham, Alabama, USA.; 18 University of Colorado Anschutz Medical Campus, Denver, Colorado, USA.; 19 Walder Foundation, Skokie, Illinois, USA.; 20 American Heart Association, Fort Worth, Texas, USA.

## Introduction

The COVID-19 pandemic has highlighted and amplified family caregiving obligations for many clinical investigators and other biomedical researchers. Unpredictable access to daycare, schools, assisted living facilities, informal networks, and other sources of care of children, older adults, or those with special needs has been harrowing. The National Academies of Science, Engineering, and Medicine emphasized such challenges will impair the vitality of the scientific workforce, calling for research and action to bolster resources for those facing family caregiving responsibilities as they pursue careers in fields that include academic medicine (1).

In the United States, where social policies addressing needs of workers with families are less robust than elsewhere in the world, engagement in demanding professional pursuits was challenging before the pandemic. Lack of family-friendly policies has a disparate impact on single parents and women, who are more likely to shoulder family caregiving responsibilities due to persistent gendered societal norms and expectations; the result is limited access of professions like medicine to the full talent pool. Prior to the pandemic, female clinician-investigators with career development awards from the NIH spent 8.5 hours more per week on parenting and domestic tasks than their male peers, after adjusting for spousal employment and other variables (2). Women from underrepresented groups may also be especially vulnerable to caregiving challenges, particularly if they have limited social and economic resources in addition to systemic barriers to participation and advancement in academics. In the decade before the pandemic, NIH data indicate women and individuals from underrepresented backgrounds disproportionately do not advance from T32 training fellowships to extramural K awards, and from K- to R-series awards (3).

Family caregiving adds to time pressures of early-career biomedical researchers. These researchers need dedicated time to collect pilot data, submit grants and publications, and garner funding to establish independent research programs (4). Limited time for key activities is demoralizing and can derail careers and thwart faculty retention by limiting publications, funding, opportunities to win awards, and promotions. Approximately 4 in 10 early-career clinician-investigators with full-time faculty appointments leave academia within 10 years due in part to time conflicts driven by caregiving (5). Underrepresented groups and women leave at higher rates than White people and men. This represents an astonishing loss of highly educated professionals from the biomedical research workforce, and reinforces inequity gaps across gender, race, and ethnicity.

## Description of the original and COVID-19 Fund to Retain Clinical Scientists Programs

### Summary of the original FRCS.

In 2015, the Doris Duke Charitable Foundation (DDCF) called for proposals from medical schools to be sites for the Fund to Retain Clinical Scientists (FRCS). The program’s primary goal was to implement and prospectively assess an intervention to retain promising early-career clinician-investigators with pressing family caregiving responsibilities (6). The intervention provided supplemental research funding to clinician-investigators demonstrating early research success and family caregiving obligations. Although such programs existed prior to 2000 (7), FRCS was the first such large-scale multicenter intervention in academic medicine.

The original FRCS supported 10 medical institutions and approximately 147 biomedical researchers prior to the pandemic. Each institution received $100,000 in direct costs, plus 8% indirect costs annually for 5 years. In 2020, 9 institutions sought renewals for 3 years with grants of annual $160,000 in direct costs per grant, plus 10% in indirect costs. Early findings indicate funds allowed recipients to repurpose their time to continue their research (4), validated the fact of caregiving demands among clinical researchers rather than reinforcing stigma (8), and normalized discussion of caregiving challenges among early-career faculty (9). Convening leaders of the 10 programs also motivated collaborative efforts to focus on challenges faced by individuals with intersecting identities from multiple marginalized groups (10).

### Summary of the COVID-19 FRCS expansion.

**Figure 1:**
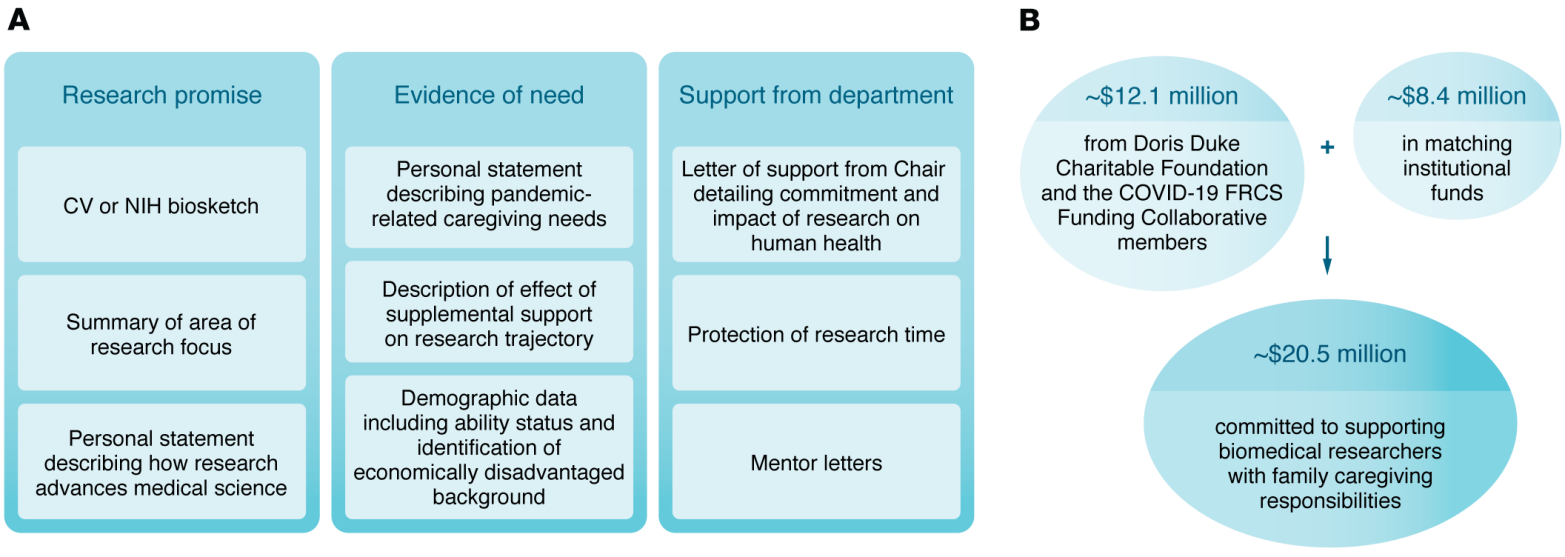
Application process and funding committed to support biomedical researchers with family caregiving responsibilities. (**A**) Application components typically included in the COVID-19 Fund to Retain Clinical Scientists (FRCS) program. Applicants must show strong evidence of research promise in an area that would advance medical science for a condition with significant clinical burden, evidence of pandemic-related family caregiving need, and commitment from their department. (**B**) Total funds committed from the Doris Duke Charitable Foundation, the COVID-19 FRCS Funding Collaborative members (American Heart Association, Burroughs Wellcome Fund, John Templeton Foundation, Rita Allen Foundation, and Walder Foundation), and matching institutional funds.

In 2021, the COVID-19 FRCS began with collaboration of DDCF with the American Heart Association, Burroughs Wellcome Fund, John Templeton Foundation, Rita Allen Foundation, and Walder Foundation. Together they issued an additional $12.1 million in October 2021 to 22 institutions, including 5 with previous FRCS program grants (Figure 1) to build on the original FRCS (4, 8–10). In addition to pursuing faculty retention and research career sustainability (including nonclinicians engaged in biomedical research, in contrast to the original FRCS), the COVID-19 FRCS seeks to elevate outstanding institutional efforts to support early-career faculty who were also navigating caregiving responsibilities during the pandemic. The new program emphasizes support for those from marginalized groups, and new grantees were identified because of their commitment to better support caregivers.

Like the original FRCS, the COVID-19–specific program aims to mitigate caregiving obligations of early-career biomedical researchers by providing extra support directly related to career development and research. For example, awardees may use the supplemental funds to hire research coordinators, biostatisticians, technicians, or administrative assistance; they may also seek grant-writing support, executive coaching, or buy-out of clinical time to devote more time to research. Funds are not for direct caregiving costs, travel, or direct research costs (which are expected to be covered by funded research grants). The program will provide funding from 2021 to 2023. The desired outcome of both the original and the COVID-19 FRCS is to mitigate factors contributing to attrition of early-career biomedical researchers while creating, strengthening, and sustaining supportive institutional cultures and programs.

## Program implementation

In December 2021, DDCF hosted a virtual conference for FRCS and COVID-19 FRCS program teams to discuss lessons learned in the original program and early experiences with the new program regarding challenges, innovations, successes, and solutions. Here, we synthesize information shared by program directors (PDs) during breakout sessions. Our primary aim is to provide the greater community of research institutions with an understanding of the programs, a conceptual framework, and strategic insights to successfully replicate the programs, given growing motivation to protect the vitality and diversity of the academic physician faculty workforce.

### Recruitment strategies.

PDs discussed strategies for advertising the COVID-19 FRCS in their institution to make potential applicants aware of the program and its goals. Many institutions used a combination of broad and targeted recruitment strategies to inform prospective applicants about the award and its ability to address challenges exacerbated by COVID-19 and caregiving responsibilities.

Most institutions have mechanisms to protect applicants’ privacy regarding the highly personal information shared in their applications and work to destigmatize caregiving in various ways to recruit applicants. Some institutions had preexisting faculty awards, monthly seminars, annual retreats, or other support systems in place for early-career investigators with caregiving responsibilities and built on these programs and culture to attract applicants without fear of stigmatization. The fact that only successful, funded investigators are eligible helps destigmatize the needs faced by early-career faculty by conveying they are normal and common. Institutions reported linking FRCS and COVID-19 FRCS programs to existing work-life integration programs and related efforts by wellness and equity or diversity programs to build camaraderie and expand peer-to-peer networking.

### Inclusion of faculty underrepresented in medicine.

Additional discussion themes included sensitivity in approaching faculty underrepresented in medicine (UriM), volatility of local epidemic conditions, and best ways to support nonprioritized applicants. Some PDs discussed challenges in reaching out to UriM faculty who may be experiencing situations that are sensitive to divulge or too complex to explain in their applications. For example, they may be experiencing economic, family, and social challenges. They may be victims of, or witnesses to, violence and structural racism and some have family or loved ones who have experienced violence or succumbed to COVID-19, which disproportionately burden communities of color. One solution proposed offering empathic communication and/or courageous conversation training to the PD or recruitment team and leveraging the assistance of the institution’s diversity office. Some PDs partnered with their institution’s diversity office to facilitate excellence in diversity, inclusion, equity, and antiracism efforts.

Another challenge discussed was that COVID-19 surges cause transient redeployment of some faculty from research activities into clinical care, as well as unpredictable closure of care facilities for children and adults. Several institutions saw increased demand for assistance, especially during times when “COVID was raging.”

### Description of the application process for faculty members.

Most PDs attempted to streamline applications to prevent additional burdens on applicants. Application components typically included are summarized in Figure 1. Institutions generally formed review committees with reviewers with experience in programs focused on supporting early-career investigators. Many included reviewers who had caregiving responsibilities themselves and/or expertise in diversity and inclusion. PDs emphasized that reviewers who are accustomed to quantitative decision making may struggle with integrating qualitative criteria, such as personal need, into decision making. One solution offered was to partition the review committee to have senior faculty review and score the research statement component, while PDs, or others, score caregiving/time burden. Including reviewers with additional perspectives and expertise in the issues faced by UriM faculty may also mitigate selection bias. Other strategies to mitigate selection bias include requiring or offering unconscious/implicit bias training to reviewers, discussing selection bias, providing equity and diversity training, and encouraging reviewers to read NIH materials on unconscious bias. Another solution offered was having applicants complete a validated questionnaire that would more uniformly assess the impact of caregiving needs.

Many PDs reported that review committees use a traditional NIH-based review process that holistically considers the research background, current support, proposed use of funds, caregiving narrative, and likelihood of research success. Some institutions simplified the process. One institution relies on only 2 scores: (a) compliance with the terms and conditions of the FRCS and (b) meeting the spirit of the FRCS. They then organize the applicants by hardship scales and rank secondarily by scientific score. Other institutions reported that scoring the caregiving responsibilities/time burdens poses a greater challenge. As one PD noted: “Given the last 2 years of COVID-19 and social stressors, there are plenty of sometimes shocking and legitimate struggles,” and this is hard to compare across applicants. The COVID-19 pandemic placed extraordinary demands on biomedical investigators, and the narratives confirm caregiving struggles were a key issue and source of distress.

### Support for faculty applicants who were not awarded.

Finally, PDs note the importance of continuing engagement with applicants who were not selected. These individuals came forward with a need and can benefit from additional support to remain in their positions or advance their careers. One potential solution is to offer those not selected for FRCS ongoing faculty development and mentoring, connections to senior faculty for advice and professional support, or professional coaching and counseling. Indeed, many funded sites complement the funding support to individual faculty from the FRCS programs with faculty development resources and programming that may be extended to faculty beyond those receiving FRCS funding.

## Evaluation efforts and anticipated impact

The original FRCS program was designed to include a formal prospective evaluation of its impact on both faculty and institutional culture. Rigorous evaluation of this initiative will play a central role in ensuring its long-term sustainability and aid others considering implementing similar initiatives.

The original FRCS-funded institutions are working together with a research team funded to collect program evaluation data from the first five cohorts of FRCS applicants. Data collection from subsequent cohorts and COVID-19 FRCS scholars will be tracked by the DDCF through annual reports. Future publications will share more about scholars’ individual experiences and the impact on their institutions in aggregate. Given current pressures faced by biomedical researchers and the evidence collected in evaluations of the program to date (4, 8, 9), FRCS is a potential exemplar for others seeking to improve equity. Leaders at unfunded institutions may find this information useful as they seek to design similar supports to enhance retention of highly trained and skilled academic faculty researchers.

Through concerted action, the academic community can address the personal needs of biomedical researchers who are also caregivers. No single barrier keeps faculty caregivers from thriving; rather, multiple obstacles and biases, some of which are unconscious, can lead to career disadvantages. Programs that address these realities should concurrently focus on reducing gender, ethnic, and racial equity gaps presenting barriers to qualified scientific minds achieving their academic potential. In addition, funded institutions will work toward sustainability of the program and unfunded institutions should encourage the leadership of their institutions to invest in similar programs. All institutions should innovate to drive cultural shifts to support biomedical faculty with family caregiving responsibilities.

## Version 1 12/01/2022

Electronic publication
